# Trait Autism is a Better Predictor of Empathy than Alexithymia

**DOI:** 10.1007/s10803-019-04080-3

**Published:** 2019-06-07

**Authors:** Punit Shah, Lucy A. Livingston, Mitchell J. Callan, Lois Player

**Affiliations:** 1 Department of Psychology, University of Bath, Bath, BA2 7AY UK; 2 Social, Genetic and Developmental Psychiatry Centre, Institute of Psychiatry, Psychology and Neuroscience, King’s College London, London, SE5 8AF UK

**Keywords:** Autism, Empathy, Alexithymia, Cognitive empathy, Affective empathy

## Abstract

It has been proposed that atypical empathy in autism spectrum disorder (ASD) is due to co-occurring alexithymia. However, difficulties measuring empathy and statistical issues in previous research raise questions about the role of alexithymia in empathic processing in ASD. Addressing these issues, we compared the associations of trait alexithymia and autism with empathy in large samples from the general population. Multiple regression analyses showed that both trait autism and alexithymia were uniquely associated with atypical empathy, but dominance analysis found that trait autism, compared to alexithymia, was a more important predictor of atypical cognitive, affective, and overall empathy. Together, these findings indicate that atypical empathy in ASD is not simply due to co-occurring alexithymia.

**Electronic supplementary material:**

The online version of this article (10.1007/s10803-019-04080-3) contains supplementary material, which is available to authorized users.

Considerable research has been directed towards studying empathy in autism spectrum disorder (ASD). Early research indicated that empathy was impaired in ASD (e.g., Baron-Cohen and Wheelwright 2004), but inconsistencies in conceptualizing and measuring empathy led to confusion in the literature (see Rogers et al. 2007). Addressing this issue has involved two particularly fruitful lines of research that we aimed to build on in the present study. First, there has been a move towards studying different components of empathy in ASD. Understanding or *knowing* what another individual is feeling (cognitive empathy) has been dissociated from *feeling* what others are feeling (affective empathy) in neuroscience and psychological research (e.g., Yang et al. 2018). Such research generally indicates that cognitive, not affective, empathy is lower in ASD (e.g., Rueda et al. 2015). Second, recent work has highlighted the role of trait alexithymia (difficulties in identifying and describing one’s own emotions) in ASD. This has taken elevated rates of alexithymia in ASD to argue that impaired emotional processing and empathy, where observed in ASD, is due to co-occurring alexithymia (e.g., Bird et al. 2010). Together, these lines of research have challenged the view that empathy is universally impaired in ASD. There are, however, several concerns with this research.

The first issue is that widely-used measures of empathy in ASD research—the Interpersonal Reactivity Index (IRI; Davis 1983) and the Empathy Quotient (Baron-Cohen and Wheelwright 2004)—were not designed to dissociate cognitive from affective empathy, and there are longstanding concerns about the IRI’s validity (see Murphy et al. 2018). To address this issue, Reniers et al. (2011) developed the Questionnaire of Cognitive and Affective Empathy (QCAE) by drawing on several empathy measures to create a more robust measure of cognitive, affective, and overall empathy. The QCAE has now been validated in several clinical and non-clinical samples (e.g., Di Girolamo et al. 2017), however, apart from one recent study (see below), it has not been used in research pertaining to ASD.

The second issue is that few studies have used appropriate analyses to investigate cognitive and affective empathy in ASD, which has contributed to the inconsistency and mixed findings in previous research (see Yang et al. 2018). Researchers have typically examined the link between one component of empathy and ASD without accounting for the other. This is problematic because affective and cognitive empathy, though conceptually distinguishable constructs, are statistically correlated and co-activated in social situations (Preckel et al. 2018). It is possible that an individual may understand what another person is feeling (i.e., have intact cognitive empathy), but only after controlling for their difficulties in feeling what that person is feeling. Equally, an individual may feel what other people are feeling (i.e., have intact affective empathy), yet experience difficulties in understanding or identifying those feelings. Therefore, investigating one component of empathy requires statistical consideration of the other during analysis‚ for a more precise understanding of (a)typical empathy.

Finally, there are methodological issues in research on the co-occurrence of ASD and alexithymia. Such research typically compares very small samples of people with and without ASD, which often lacks sufficient statistical power to test the unique associations of trait autism and alexithymia (see also, Nicholson et al. 2018). Crucially, this research also involves matching groups with and without ASD on alexithymia, with studies reporting no association between ASD and atypical empathy after controlling for alexithymia (e.g., Bird et al. 2010). However, because the prevalence of alexithymia is much lower in typically developing compared to autistic populations (5% vs 50%, respectively; Kinnaird et al. 2019), matching groups for alexithymia is potentially problematic. Matching groups in this way necessitates biased sampling, therefore neither group is representative of autistic or typically developing populations (see also, Lassalle et al. 2019), resulting in inappropriate statistical group comparisons and potentially inaccurate population-level inferences. For example, Oakley et al. (2016), when investigating a small sample of 19 autistic and 23 non-autistic adults (matched for alexithymia), found that affective impairments were solely associated with alexithymia, whereas theory of mind (analogous to cognitive empathy) was only impaired in ASD. However, Oakley et al. did not statistically control for theory of mind when examining the associations between ASD, alexithymia and affective processing, nor control for affective processing when measuring the links between ASD, alexithymia and theory of mind. Given the small sample size, it is also unclear whether ASD has no association with affective impairments over and above alexithymia, or whether previous findings have been Type II errors due to suboptimal statistical analysis. Furthermore, it is questionable whether their statistical inferences—i.e., generalizing from samples to the population—were appropriate due to the biased sampling required for alexithymia-matched groups. Notwithstanding these concerns, previous research leads to testable predictions that trait autism (hereafter ‘autism’), not alexithymia, should be associated with low cognitive empathy, whereas alexithymia, not autism, should be related to low affective empathy. These hypotheses are explored in the present research.

To our knowledge, only one study has explored the relationship between ASD and alexithymia, and their relative associations with cognitive and affective empathy. Mul et al. (2018), in a small study of adults with (*n* = 26) and without (*n* = 26) ASD, found that alexithymia partially mediated the links between ASD and both low cognitive and affective empathy. Although this supports the idea that alexithymia may partly contribute to atypical empathy in ASD, it does not support claims (i.e., Bird and Cook 2013) that empathy impairments, where observed in ASD, are entirely due to alexithymia. Moreover, Mul et al. noted the small sample in their study as a limitation, which also resulted in limited variance in alexithymia in the control compared to the ASD group. In addition, the relationships of ASD and alexithymia, separately with cognitive and affective empathy, were not examined whilst accounting for the other component of empathy in their mediation analyses. Mul et al.’s study was also not designed to compare the statistical importance of ASD and alexithymia as predictors of atypical empathy.

In view of the limitations of previous research, we suggest that the extent to which ASD and alexithymia are related to empathy requires further investigation. Addressing concerns with previous work, we designed studies using the QCAE and measures of trait autism and alexithymia in two large community samples drawn from the general population. Despite potential limitations with this approach (see “Discussion”), this avoided inappropriate comparisons of small and biased samples of adults with and without ASD (matched for alexithymia), and poorly powered statistical analyses, commonly found in previous research. Instead, we aimed to conduct the most well-powered statistical examination of the interrelationships between autism, alexithymia, and different components of empathy to date. Specifically, we compared the associations of autism and alexithymia with overall empathy, and each component of empathy whilst controlling for the other component. Critically, we compared the statistical importance of autism and alexithymia as predictors of empathy by using dominance analysis for the first time in this field of research.

## Methods

### Participants, Measures, and Procedure

Participants formed a community sample drawn from online sources of 306 adults (45% female), aged between 18 and 85 years (*M* = 34.0 years, *SD* = 11.9 years). A power analysis (Faul et al. 2007) revealed that we had 95% power to detect “small-to-medium” unique associations in our regression analyses (*f*^2^ = 0.03, *α* = 0.05, 2-tailed).^1^ Participants completed self-report measures of trait autism (28-item Short Autism-Spectrum Quotient, AQS; Hoekstra et al. 2011), alexithymia (20-item Toronto Alexithymia Scale, TAS-20; Bagby et al. 1994), and empathy (31-item Questionnaire of Cognitive and Affective Empathy, QCAE; Reniers et al. 2011). The AQS, measuring (dis)agreement with statements on autism-like symptoms on a 4-point Likert scale, is a validated and widely used quantitative measure of autistic traits; scores range between 28 (few autistic traits) and 112 (many autistic traits). It has been shown to measure the same latent construct in adults with and without a clinical diagnosis of ASD (Murray et al. 2014) and in males and females (Grove et al. 2017). The TAS-20, measuring (dis)agreement with statements about difficulties identifying and describing one’s own emotions on a 5-point Likert scale, quantified alexithymia; scores range between 20 (low alexithymia) and 100 (high alexithymia). The TAS-20 has been used extensively in samples with and without ASD, notably to test the competing influences of autism and alexithymia on psychological variables (e.g., Shah et al. 2016a, b), as in the current study. The QCAE measured (dis)agreement with statements about understanding others’ feelings and feeling others’ feelings on a 4-point Likert scale; scores for overall empathy range between 31 (low empathy) and 124 (high empathy), cognitive empathy between 19 and 76, and affective empathy between 12 and 48. The QCAE has also been validated and used widely to measure (a)typical levels of empathy (see Lockwood 2016). All measures had good reliability in the current study (AQS: *α* = .82, TAS-20: *α* = .90, QCAE: *α* = .91). The questionnaires were presented in a randomized order, followed by questions about age and sex.

## Results

A wide range of autism and alexithymia scores were present in the sample (Table 1), confirming adequate variance in line with previous research (e.g., Farmer et al. 2017; Shah et al. 2016a). All variables were moderately correlated (Table 1). Notably, both autism and alexithymia were negatively correlated with cognitive, affective, and overall empathy. There were positive correlations between autism and alexithymia, and between cognitive and affective empathy. Male participants also reported lower levels of empathy than females (Online Resource—Table 1). Multiple regression analyses measured the unique associations of autism and alexithymia with (i) overall empathy, (ii) cognitive empathy, and (iii) affective empathy. Sex was included in all regressions, given the sex differences in empathy. Results showed that autism and alexithymia were both significant predictors of low (i) overall empathy, (ii) cognitive empathy after accounting for affective empathy, whereas (iii) alexithymia, not autism, was associated with higher affective empathy after accounting for cognitive empathy (see Table 2).

**Table 1:** Means and correlations

Measure	*M* (*SD*)	1	2	3	4
1. Trait autism (AQS)	65.08 (10.27)	–			
2. Trait alexithymia (TAS-20)	47.47 (14.03)	.46***	–		
3. Cognitive empathy (QCAE cognitive subscale)	57.94 (8.54)	− .50***	− .44***		
4. Affective empathy (QCAE affective subscale)	33.68 (5.70)	− .26***	− .19**	.51***	
5. Overall empathy (QCAE overall score)	91.62 (12.45)	− .46***	− .39***	.92***	.81***

Trait autism was measured using the 28-item Short Autism-Spectrum Quotient (AQS; Hoekstra et al. 2011), alexithymia using the 20-item Toronto Alexithymia Scale (TAS-20; Bagby et al. 1994), and empathy using cognitive and affective subscales and overall scores of the Questionnaire of Cognitive and Affective Empathy (QCAE; Reniers et al. 2011)

**p < .01

***p < .001

**Table 2:** Regression and dominance analyses for overall, cognitive and affective empathy

Predictor	*β*	*t*	*p*	*sr* ^2^	GDW
(i) Overall empathy—*F*(3, 302) = 39.22, *R*^2^ = 0.28, *p *< .001					
Sex (1 = male, 0 = female)	− .18	− 3.64	< .001	0.042	0.050
Autism	− .34	− 6.26	< .001	0.115	0.147
Alexithymia	− .19	− 3.33	.001	0.035	0.084
(ii) Cognitive empathy—*F*(4, 301) = 65.99, *R*^2^ = 0.47, *p* < .001					
Affective empathy	.46	9.66	< .001	0.236	0.199
Sex	.17	3.60	< .001	0.041	0.014
Autism	− .28	− 5.82	< .001	0.101	0.143
Alexithymia	− .26	− 5.49	< .001	0.091	0.053
(iii) Affective empathy—*F*(4, 301) = 50.08, *R*^2^ = 0.40, *p *< .001					
Cognitive empathy	.52	9.66	< .001	0.236	0.212
Sex	− .38	− 8.34	< .001	0.187	0.148
Autism	− .004	− 0.08	.94	0.00003	0.025
Alexithymia	.13	2.48	.014	0.020	0.014

Examination of VIF values across the regression analyses indicated that multicollinearity was not a concern (all < 10), and the residuals were normally distributed. Durbin–Watson statistics were inspected and found to be ~ 2 across the regression analyses, suggesting that errors were uncorrelated and thus independent. Together, the data were suitable for multiple linear regression analysis

*Β* standardized regression coefficient, *t* Student’s t-statistic, *p* p value, *sr*^2^ semi-partial correlation squared, *GDW* General Dominance Weight (higher GDW values indicate a more important predictor)

Multicollinearity was not a concern as the variables were moderately correlated (Table 1), in line with research finding that autism, alexithymia, and empathy are different constructs. Equally, however, given the finding that autism and alexithymia were both significantly associated with atypical empathy, it was not appropriate to determine the relative *importance* of each predictor by simply comparing the size of their beta coefficients (see Budescu 1993). To overcome this problem, we employed dominance analysis, which involves computing each predictor’s incremental validity (or semi-partial correlation squared, *sr*^2^) across all possible subset regression models involving that predictor. These validities are then used to establish the relative importance of each predictor to the criterion, yielding General Dominance Weights (GDW), which represent the average *sr*^2^ across submodels for a given predictor. The GDW sum to the overall model *R*^2^ for a given criterion and are used to rank-order each predictor’s relative importance to the criterion. In other words, dominance analysis permits the ranking of statistical *importance*, which is not possible through conventional regression (see Nimon and Oswald 2013). Including participant sex in the models, we performed dominance analyses using the *yhat* package in R (Nimon et al. 2013). Results showed that autism dominated alexithymia as a predictor of cognitive, affective, and overall empathy (Table 2). Further, bootstrapping (1000 resamples) estimated reproducibility rates (RR) of how likely the dominance relationship would be observed in the population from how often it occurred in the bootstrapped samples. This showed that autism dominated alexithymia for overall (RR = 90.5%), cognitive (RR = 75.1%), and affective (RR = 79.6%) empathy. RRs ≥ 70% indicate high confidence that the dominance relationship observed in the sample would exist in the population (Azen 2013).

### Exploratory Analyses and Replication Study

Exploratory analyses^2^ showed that males reported significantly more autistic and alexithymic traits than female participants (Online Resource—Table 1). We also explored whether the associations between autism and empathy, and alexithymia and empathy, were moderated by sex by including sex × autism and sex × alexithymia interaction terms in the original regression analyses. These interaction terms were not statistically significant predictors of overall and cognitive empathy scores (Online Resource—Tables 2, 3). However, there was a statistically significant sex × alexithymia interaction for affective empathy (Online Resource—Table 4). Simple slopes analysis revealed that an association between alexithymia and higher affective empathy was significant in male (*β* = 0.24, *t* = 3.40, *p* < .001) but not female (*β* = − 0.02, *t* = − 0.27, *p* = .79) participants.

Following recommendations to improve the replicability of clinical psychological science (Tackett et al. 2017), we conducted a replication study in another large sample that completed the same procedure (see Online Resource—Replication Study). Data were submitted to regression analyses (see Table 3) to measure the unique associations of autism and alexithymia with (i) overall, (ii) cognitive, and (iii) affective empathy. Sex, sex × autism, and sex × alexithymia were included in all models. Replicating the original findings, autism and alexithymia were (i) unique and significant predictors of low overall empathy, and (ii) unique and significant predictors of low cognitive empathy whilst accounting for affective empathy. In contrast to the original result, (iii) *autism*, but not alexithymia, was associated with higher affective empathy whilst accounting for cognitive empathy. In line with the original study, the sex × alexithymia interaction was statistically significant, and simple slopes analysis revealed that the association between alexithymia and higher affective empathy was significant for male (*β* = 0.20, *t* = 2.50, *p* = .013) but not female (*β* = − 0.05, *t* = − 0.67, *p* = .51) participants. Replicating the original dominance analysis, General Dominance Weights (GDW) and Reproducibility Rates (RR) indicated that autism dominated alexithymia as a predictor of overall (GDW autism = 0.10, alexithymia = 0.07; RR = 75.9%), cognitive (GDW autism = 0.16, alexithymia = 0.11; RR = 85.3%), and affective (GDW autism = 0.007, alexithymia = 0.005; RR = 79.4%) empathy. Together, the original pattern of results was replicated; that is, autism was a better predictor of empathy than alexithymia.

**Table 3:** Replication study—regression analyses for overall, cognitive and affective empathy

Predictor	*β*	*t*	*p*
(i) Overall empathy—*F*(5, 348) = 30.28, *R*^2^ = 0.30, *p *< .001			
Sex (1 = male, 0 = female)	− .34	− 7.48	< .001
Sex × autism	.02	0.30	.77
Sex × alexithymia	.10	1.95	.052
Autism	− .25	− 4.72	< .001
Alexithymia	− .18	− 3.41	.001
(ii) Cognitive empathy–*F*(6, 347) = 40.61, *R*^2^ = 0.41, *p *< .001			
Affective empathy	.31	6.82	< .001
Sex	− 1.62	− 1.62	.11
Sex × autism	.03	0.56	.57
Sex × alexithymia	.01	0.10	.92
Autism	− .34	− 6.96	< .001
Alexithymia	− .23	− 4.74	< .001
(iii) Affective empathy—*F*(6, 347) = 22.81, *R*^2^ = 0.28, *p *< .001			
Cognitive empathy	.38	6.82	< .001
Sex	− .33	− 6.99	< .001
Sex × autism	− .02	− 0.28	.078
Sex × alexithymia	.12	2.29	.023
Autism	.12	2.03	.044
Alexithymia	.07	1.19	.24

Examination of VIF values across the regression analyses indicated that multicollinearity was not a concern (all < 10), and the residuals were normally distributed. Durbin–Watson statistics were inspected and found to be ~ 2 across the regression analyses, suggesting that errors were uncorrelated and thus independent. Together, the data were suitable for multiple linear regression analysis

*Β* Standardized regression coefficient, *t* Student’s t-statistic, *p* p value

## Discussion

The association between autism and alexithymia, the link between cognitive and affective empathy, and sex differences in empathy are in line with previous research. These results support theories that participant sex (Baron-Cohen and Wheelwright 2004; Greenberg et al. 2018) and alexithymia (Bird and Cook 2013) are broadly relevant to understanding empathy in ASD. Our results also support claims that, although partly dissociable, cognitive and affective empathy are overlapping constructs (Preckel et al. 2018). We therefore examined overall empathy (combining cognitive and affective scores) as the starting point in our multivariate analyses. Across both studies, we found that, although alexithymia partly contributes to low empathy (in line with Mul et al. 2018), autism is more predictive of low overall empathy in the population. Critically, the consistency of the dominance analyses across both studies highlighted the greater statistical importance of autism as a predictor of low overall empathy when compared to alexithymia. Although dominance analysis only provides metrics for *statistical importance*, we suggest that our results support claims that low empathy is a *clinically important* feature of ASD. The present study is of course not sufficient to substantiate this proposal, however it does provide fresh evidence in support of longstanding (e.g., Baron-Cohen and Wheelwright 2004) and recent (Russ et al. 2018) proposals that measuring overall empathy has utility in the diagnosis and management of ASD (see also, Robinson and Elliott 2016). Building on this previous work, we propose that measuring and managing trait autism, compared to alexithymia, is likely to be a more efficacious approach to investigate and ameliorate empathy-related difficulties in ASD.

Given evidence for the dissociation between cognitive and affective empathy (e.g., Reniers et al. 2011), our analyses of these subcomponents provide a more precise understanding of empathy in relation to autism. Across both studies, autism and alexithymia were both uniquely associated with difficulties in *knowing* what people are feeling (i.e., low cognitive empathy), whilst controlling for difficulties in *feeling* what others are feeling (i.e., low affective empathy). However, we note that, although alexithymia partly contributed to atypical empathy, autism was more predictive of lower cognitive empathy. Accordingly, autism was, across all dominance analyses, a far more important predictor of low cognitive empathy than alexithymia. This is consistent with findings that ASD is characterized by poor cognitive empathy (e.g., Rueda et al. 2015) and, importantly, the present study is the first to demonstrate the robustness of the association between autism and low cognitive empathy even after accounting for alexithymia and affective empathy.

Our results also fit with previous evidence for a link between alexithymia and impaired cognitive empathy (e.g., Di Girolamo et al. 2017; Moriguchi et al. 2006) and reports that emotional awareness is associated with cognitive empathy or theory of mind (Lane et al. 2015). However, unlike previous research, this is the first study to detect a relationship between alexithymia and low cognitive empathy even after controlling for autism and affective empathy. These results are therefore not consistent with recent evidence that alexithymia is unrelated to theory of mind (synonymous with cognitive empathy; Rueda et al. 2015) after accounting for autism (i.e., Oakley et al. 2016). This may be due to the fact that behavioral, instead of questionnaire, measures were used in recent research, and/or because previous studies did not control for affective empathy, and/or have the statistical power to detect this pattern of results. It is also debated whether ‘cognitive empathy’ and ‘theory of mind’ are synonymous because it remains unclear whether experimental tasks of these abilities are measuring the same construct (Happé et al. 2017; Warrier and Baron-Cohen 2018). We therefore suggest that, following refinement of the theoretical overlap, terminology, and measures of these social cognitive processes (Happé et al. 2017), it may be necessary to conduct a follow-up of the present study that includes refined measures of cognitive empathy and theory of mind (see Livingston et al. 2019).

Our findings on affective empathy were novel but inconsistent. The first regression analysis unexpectedly showed that alexithymia, but not autism, was associated with high affective empathy whilst controlling for cognitive empathy. These results fit with research showing that alexithymia may be associated with high levels of affective empathy (Guttman and Laporte 2002). However, the second regression analysis failed to replicate this finding, instead showing that autism, not alexithymia, was associated with high affective empathy. Moreover, although autism was not a *significant* predictor of affective empathy in the first regression model including other predictor variables (sex, cognitive empathy, alexithymia), it was still a more *important* predictor of affective empathy than alexithymia as dominance analysis averages across *all possible subset regression models* involving autism. The second regression was, however, consistent with the dominance analysis; autism emerged as a statistically significant and more important predictor of affective empathy. Together, dominance analysis, across both studies, demonstrated that autism was a better predictor of higher affective empathy compared to alexithymia. This result is consistent with research that autism, but not alexithymia, may be associated with a hypersensitivity to others’ feelings (Fan et al. 2014; Smith 2009). Finally, additional exploratory analyses revealed that the association between alexithymia and higher affective empathy, evident in both our studies, was only found in male, and not female, participants. This is somewhat consistent with research on the association between alexithymia and empathy (after accounting for autism), which has typically been found in all male samples (e.g., Bird et al. 2010).

Despite some of these interesting and potentially important findings regarding affective empathy, the inconsistencies within our study and in previous research indicates that it is most prudent to conclude that neither autism, nor alexithymia, are robust predictors of atypical affective empathy, especially when compared to their stronger associations with participant sex and cognitive empathy. This is supported by emerging evidence that, in clinically diagnosed people with ASD, neither autism, nor alexithymia, are associated with affective empathy (Ziermans et al. 2018). Our data are therefore not consistent with claims that atypical affective empathy, where observed in ASD, is due to alexithymia (Bird et al. 2010). Rather, a growing body of research indicates that alexithymia-based explanations for the link between autism and atypical empathy (e.g., Bird and Cook 2013) may have been overstated in previous research. Overall, therefore, we suggest that the interrelationships between sex, autism, alexithymia, and affective empathy are more complex than previously reported and will require further investigation in future research (see also, Lassalle et al. 2019).

### Strengths, Limitations, and Future Directions

The current study indicates that greater consideration of participant sex will be required in future research on the competing influences of autism and alexithymia on atypical empathy (see also, Greenberg et al. 2018). It seems possible that if previous studies on alexithymia-based explanations of empathic difficulties in ASD (e.g., Bird et al. 2010) were conducted in samples containing females, a different pattern of results would emerge. More broadly, there is growing evidence for sex differences in social-emotional processing in people with ASD (see Lai et al. 2015, for overview; Livingston and Happé 2017). Therefore, studies with large and representative samples of males and females will, in future, be required to investigate sex-specific empathy profiles (if any) in ASD.

Further research will also be necessary to overcome several other limitations of the current study. The present study was conducted in large samples drawn from the general population to enable high-powered analyses that are currently not possible to perform with equal rigor in clinical samples due to practical considerations. Well-powered statistical analyses allowed us to find new evidence in support of existing clinical research, and detect novel associations that will inform the design of future research in clinically diagnosed people with ASD. However, while the study of autistic traits is widely used to inform understanding of autism (see Ruzich et al. 2015), there are many limitations and ongoing debates about the appropriateness of measuring sub-clinical autistic traits as a proxy for understanding clinically diagnosed ASD. For example, it remains debated whether ASD and population-level autism traits lie on a quantitative continuum or are qualitatively distinct (e.g., Constantino and Charman 2016; Frazier et al. 2010; Volkmar and McPartland 2016). It will therefore be critically important to re-examine alexithymia’s role in empathy in clinically diagnosed people with ASD, and it is hoped that the current study provides the impetus for such future research.

We used a well-validated empathy questionnaire to collect a large dataset to avoid problems with experimental tasks of empathy (see Mackes et al. 2018, for recent discussion), such as their lack of ecological validity and narrow conceptualizations of empathy (e.g., empathy for pain; see Lockwood 2016). However, it will be important to determine whether our results can be reproduced using new video-based experimental measures of empathy that are currently undergoing development (e.g., Mackes et al. 2018). Moving forward, we propose that studies combining appropriate experimental and questionnaire measures, in large samples, will be required to further elucidate the role of alexithymia (if any) in atypical empathy in ASD. Finally, our research, following previous studies in this field, was cross-sectional. Longitudinal research will of course be required to examine whether atypical emotional processing and empathy in ASD is a cause or consequence of alexithymia or other co-occurring traits (Livingston and Livingston 2016; Poquérusse et al. 2018).

## Conclusions

To conclude, the present study indicates that trait autism, compared to alexithymia, is a better predictor of cognitive, affective, and overall empathy. We suggest that the role of alexithymia in empathic processing in ASD requires further investigation before considering any clinical implications arising from such research. To this end, we call for additional research using (i) appropriately large and representative samples of male and female individuals with ASD, (ii) new and improved experimental and questionnaire measures of empathy, and (iii) well-powered multivariate analyses as used in the present study, for a re-examination of the role of alexithymia in empathic processing in ASD.

## Electronic supplementary material

Below is the link to the electronic supplementary material.

Supplementary material 1 (DOCX 32 kb)

## Footnotes

1 Fourteen additional participants were recruited but excluded from the final sample. Four participants failed to complete the study and 3 participants completed the study twice. Seven participants were excluded as they were multivariate outliers with residuals more than 3SDs from the mean in the regression analyses.

2 We thank an anonymous reviewer for suggesting these analyses.
