# Supplementary material for: Trait Autism is a Better Predictor of Empathy than Alexithymia
Source: J Autism Dev Disord. 2019 Jun 7;49(10):3956–64. doi: 10.1007/s10803-019-04080-3 (PMC6751139; doi:10.1007/s10803-019-04080-3)
Supplement: Supplementary file 1 — Supplementary material 1 (DOCX 32 kb) [file 10803_2019_4080_MOESM1_ESM.docx]

**Online Resource 1 – Supplemental Material**

| **Table 1 – Sex Differences** | | | | | | | | | | | | | |
| --- | --- | --- | --- | --- | --- | --- | --- | --- | --- | --- | --- | --- | --- |
|  | | ***t*** | | **df** | | ***p*** | | **Mean Difference** | | **SE Difference** | | **Cohen's *d*** | |
| Autism |  | -2.629 |  | 304.0 |  | .009 |  | -3.070 |  | 1.168 |  | -0.302 |  |
| Alexithymia |  | -4.157 |  | 304.0 |  | < .001 |  | -6.525 |  | 1.569 |  | -0.477 |  |
| Overall Empathy |  | 5.038 |  | 304.0 |  | < .001 |  | 6.930 |  | 1.375 |  | 0.578 |  |
| Cognitive Empathy |  | 2.207 |  | 304.0 |  | .028 |  | 2.151 |  | 0.975 |  | 0.253 |  |
| Affective Empathy |  | 8.030 |  | 304.0 |  | < .001 |  | 4.779 |  | 0.595 |  | 0.922 |  |
|  | | | | | | | | | | | | | |
| *Note.  t* = Student's t-test. | | | | | | | | | | | | | |

**Table 2 – Exploratory Overall Empathy Regression Model**

| **Model Summary** | | | | | | | | | | |  |  |  |  |  |  |
| --- | --- | --- | --- | --- | --- | --- | --- | --- | --- | --- | --- | --- | --- | --- | --- | --- |
| **Model** | | **R** | | **R²** | | **Adjusted R²** | | **RMSE** | | |  |  |  |  |  |  |
| 1 |  | 0.534 |  | 0.285 |  | 0.273 |  | 0.853 | |  |  |  |  |  |  |  |
|  | | | | | | | | | | |  |  |  |  |  |  |
| **ANOVA** | | | | | | | | | | | | | | | | |
| **Model** | |  | | | **Sum of Squares** | | | | **df** | | **Mean Square** | | ***F*** | | ***p*** | |
| 1 |  | Regression | |  | 86.92 | | |  | 5 |  | 17.384 |  | 23.91 |  | < .001 |  |
|  |  | Residual | |  | 218.08 | | |  | 300 |  | 0.727 |  |  |  |  |  |
|  |  | Total | |  | 305.00 | | |  | 305 |  |  |  |  |  |  |  |
|  | | | | | | | | | | | | | | | | |

| **Coefficients** | | | | | | | | | | | | | |
| --- | --- | --- | --- | --- | --- | --- | --- | --- | --- | --- | --- | --- | --- |
| **Model** | |  | | **b** | | **SE** | | **β** | | ***t*** | | ***p*** | |
| 1 |  | (Intercept) |  | -0.008 |  | 0.050 |  |  |  | -0.152 |  | .879 |  |
|  |  | Autism |  | -0.366 |  | 0.059 |  | -0.366 |  | -6.183 |  | < .001 |  |
|  |  | Alexithymia |  | -0.183 |  | 0.057 |  | -0.183 |  | -3.221 |  | .001 |  |
|  |  | Sex*Alexithymia |  | 0.072 |  | 0.057 |  | 0.070 |  | 1.273 |  | .204 |  |
|  |  | Sex*Autism |  | -0.061 |  | 0.057 |  | -0.062 |  | -1.069 |  | .286 |  |
|  |  | Sex |  | -0.179 |  | 0.050 |  | -0.179 |  | -3.561 |  | < .001 |  |
|  | | | | | | | | | | | | | |

**Table 3 – Exploratory Cognitive Empathy Regression Model**

| **Model Summary** | | | | | | | | | | |  |  |  |  |  |  |
| --- | --- | --- | --- | --- | --- | --- | --- | --- | --- | --- | --- | --- | --- | --- | --- | --- |
| **Model** | | **R** | | **R²** | | **Adjusted R²** | | **RMSE** | | |  |  |  |  |  |  |
| 1 |  | 0.688 |  | 0.473 |  | 0.462 |  | 0.733 | |  |  |  |  |  |  |  |
|  | | | | | | | | | | |  |  |  |  |  |  |
| **ANOVA** | | | | | | | | | | | | | | | | |
| **Model** | |  | | | **Sum of Squares** | | | | **df** | | **Mean Square** | | ***F*** | | ***p*** | |
| 1 |  | Regression | |  | 144.2 | | |  | 6 |  | 24.039 |  | 44.71 |  | < .001 |  |
|  |  | Residual | |  | 160.8 | | |  | 299 |  | 0.538 |  |  |  |  |  |
|  |  | Total | |  | 305.0 | | |  | 305 |  |  |  |  |  |  |  |
|  | | | | | | | | | | | | | | | | |

| **Coefficients** | | | | | | | | | | | | | |
| --- | --- | --- | --- | --- | --- | --- | --- | --- | --- | --- | --- | --- | --- |
| **Model** | |  | | **b** | | **SE** | | **β** | | ***t*** | | ***p*** | |
| 1 |  | (Intercept) |  | 0.018 |  | 0.043 |  |  |  | 0.414 |  | .679 |  |
|  |  | Autism |  | -0.299 |  | 0.052 |  | -0.299 |  | -5.772 |  | < .001 |  |
|  |  | Alexithymia |  | -0.251 |  | 0.049 |  | -0.251 |  | -5.153 |  | < .001 |  |
|  |  | Sex*Alexithymia |  | -0.049 |  | 0.049 |  | -0.048 |  | -1.002 |  | .317 |  |
|  |  | Sex*Autism |  | -0.043 |  | 0.049 |  | -0.044 |  | -0.882 |  | .379 |  |
|  |  | Sex |  | 0.169 |  | 0.047 |  | 0.169 |  | 3.599 |  | < .001 |  |
|  |  | Affective Empathy |  | 0.466 |  | 0.048 |  | 0.466 |  | 9.753 |  | < .001 |  |
|  | | | | | | | | | | | | | |

**Table 4 – Exploratory Affective Empathy Regression Model**

| **Model Summary** | | | | | | | | | | |  |  |  |  |  |  |
| --- | --- | --- | --- | --- | --- | --- | --- | --- | --- | --- | --- | --- | --- | --- | --- | --- |
| **Model** | | **R** | | **R²** | | **Adjusted R²** | | **RMSE** | | |  |  |  |  |  |  |
| 1 |  | 0.644 |  | 0.414 |  | 0.402 |  | 0.773 | |  |  |  |  |  |  |  |
|  | | | | | | | | | | |  |  |  |  |  |  |
| **ANOVA** | | | | | | | | | | | | | | | | |
| **Model** | |  | | | **Sum of Squares** | | | | **df** | | **Mean Square** | | ***F*** | | ***p*** | |
| 1 |  | Regression | |  | 126.3 | | |  | 6 |  | 21.055 |  | 35.24 |  | < .001 |  |
|  |  | Residual | |  | 178.7 | | |  | 299 |  | 0.598 |  |  |  |  |  |
|  |  | Total | |  | 305.0 | | |  | 305 |  |  |  |  |  |  |  |
|  | | | | | | | | | | | | | | | | |

| **Coefficients** | | | | | | | | | | | | | |
| --- | --- | --- | --- | --- | --- | --- | --- | --- | --- | --- | --- | --- | --- |
| **Model** | |  | | **b** | | **SE** | | **β** | | ***t*** | | ***p*** | |
| 1 |  | (Intercept) |  | -0.029 |  | 0.046 |  |  |  | -0.631 |  | .529 |  |
|  |  | Autism |  | -0.002 |  | 0.058 |  | -0.002 |  | -0.029 |  | .977 |  |
|  |  | Alexithymia |  | 0.120 |  | 0.053 |  | 0.120 |  | 2.260 |  | .025 |  |
|  |  | Sex*Alexithymia |  | 0.129 |  | 0.052 |  | 0.125 |  | 2.509 |  | .013 |  |
|  |  | Sex*Autism |  | -0.008 |  | 0.052 |  | -0.008 |  | -0.157 |  | .876 |  |
|  |  | Sex |  | -0.376 |  | 0.046 |  | -0.376 |  | -8.228 |  | < .001 |  |
|  |  | Cognitive Empathy |  | 0.518 |  | 0.053 |  | 0.518 |  | 9.753 |  | < .001 |  |
|  | | | | | | | | | | | | | |

**Replication Study**

Participants formed a sample drawn from online sources of 354 adults (53% female) aged between 18 and 81 years (*M* = 36.32 years, *SD* = 12.05 years). A power analysis (Faul, Erdfelder, Lang, & Buchner, 2007) revealed that we had at least 95% power to detect “small-to-medium” unique associations in our regression analyses (*f*^2^ = 0.03, *α* = 0.05, 2-tailed). Ten additional participants were recruited but excluded from the final sample. Seven participants failed to complete the study. Three participants were excluded as they were multivariate outliers with residuals more than 3SDs from the mean in all the regression analyses. The questionnaire measures were identical to those used in the original study, and presented to participants in a randomized order, followed by questions about their age and sex. A wide range of autism (*M* = 65.41, *SD* = 9.98), alexithymia (*M* = 48.38, *SD* = 13.87), cognitive empathy (*M* = 58.13, *SD* = 9.15), affective empathy (*M* = 33.45, *SD* = 6.26), and overall empathy (*M* = 91.68, *SD* = 12.94) scores were present in line with the original study. Data analysis focused on (multivariate) regression and dominance analysis with a view to test whether the pattern of results from the original study – i.e., that autism is a better predictor of atypical empathy then alexithymia – could be replicated.
